# Visual features are processed before navigational affordances in the human brain

**DOI:** 10.1038/s41598-024-55652-y

**Published:** 2024-03-06

**Authors:** Kshitij Dwivedi, Sari Sadiya, Marta P. Balode, Gemma Roig, Radoslaw M. Cichy

**Affiliations:** 1 Department of Education and Psychology, Freie Universität Berlin, Berlin, Germany; 2 Department of Computer Science, Goethe University Frankfurt, Frankfurt, Germany; 3 Institute of Neuroinformatics, ETH Zurich and University of Zurich, Zurich, Switzerland; 4 Frankfurt Institute for Advanced Studies (FIAS), Frankfurt, Germany; 5 The Hessian Center for Artificial Intelligence (hessian.AI), Darmstadt, Germany

**Keywords:** Neuroscience, Psychology

## Abstract

To navigate through their immediate environment humans process scene information rapidly. How does the cascade of neural processing elicited by scene viewing to facilitate navigational planning unfold over time? To investigate, we recorded human brain responses to visual scenes with electroencephalography and related those to computational models that operationalize three aspects of scene processing (2D, 3D, and semantic information), as well as to a behavioral model capturing navigational affordances. We found a temporal processing hierarchy: navigational affordance is processed later than the other scene features (2D, 3D, and semantic) investigated. This reveals the temporal order with which the human brain computes complex scene information and suggests that the brain leverages these pieces of information to plan navigation.

## Introduction

By looking even only briefly at a scene, we rapidly extract multifaceted pieces of visual information^1–4^ that enable us to navigate through the scene, for example by planning a route through it. How does the brain compute visual information that affords navigational route planning in a scene?

This fundamental question has been the subject of considerable debate. On the one hand, previous research indicates that navigational affordance is deeply intertwined with even low level visual features^5–7^. This suggests that early affordance processes happen in parallel and can influence scene perception^6^. On the other hand, navigation can be conceived as a complex computational feat that integrates several different scene features that need to be computed first, including 3-dimensional and semantic scene aspects^8,9^. For instance, successfully navigating the immediate environment requires localizing obstacles and finding out a way around them, which necessitates 3D scene information. Similarly, semantic scene classification may benefit route planning as navigating typical basements, balconies, and garages require different procedures. Finally, research focusing on object affordance has demonstrated that these affordances are results of expectation and meaning and are thus secondary to perception^8,9^. To investigate how these cognitive processes relate to each other we explore the temporal order in which cognitive representations capturing affordance and other visual features emerge. Specifically, we hypothesize that representation of navigational affordances emerges later in time than representations that capture other visual features such as 2D, 3D and semantic information.

To test the hypothesis, we collected human electroencephalography (EEG) responses to indoor scene images, capturing the temporal order of scene feature processing in the human brain during visual scene perception.

We investigated three types of visual features: 2-dimensional (2D), 3-dimensional (3D) and semantic features. We operationalized the visual features in the indoor scene images as activations of deep neural networks (DNNs) trained to perform respective 2D, 3D and semantic tasks^10^. Navigational features are captured using navigational affordance maps (NAM) constructed using human behavioral responses when planning exit routes in natural indoor scene images^11^.

We then related the visual and navigational features to EEG data using representational similarity analysis (RSA)^26^ in a time-resolved manner, yielding time courses with which visual representations of particular features emerge. Finally, we compared these features and NAM with EEG, revealing the temporal order in which these features are processed in the human brain.

We found that navigational affordance representations emerged significantly later than visual features. This is consistent with the view that the brain uses 2D, 3D and semantic scene features to facilitate navigation planning.

## Results

**Figure 1:**
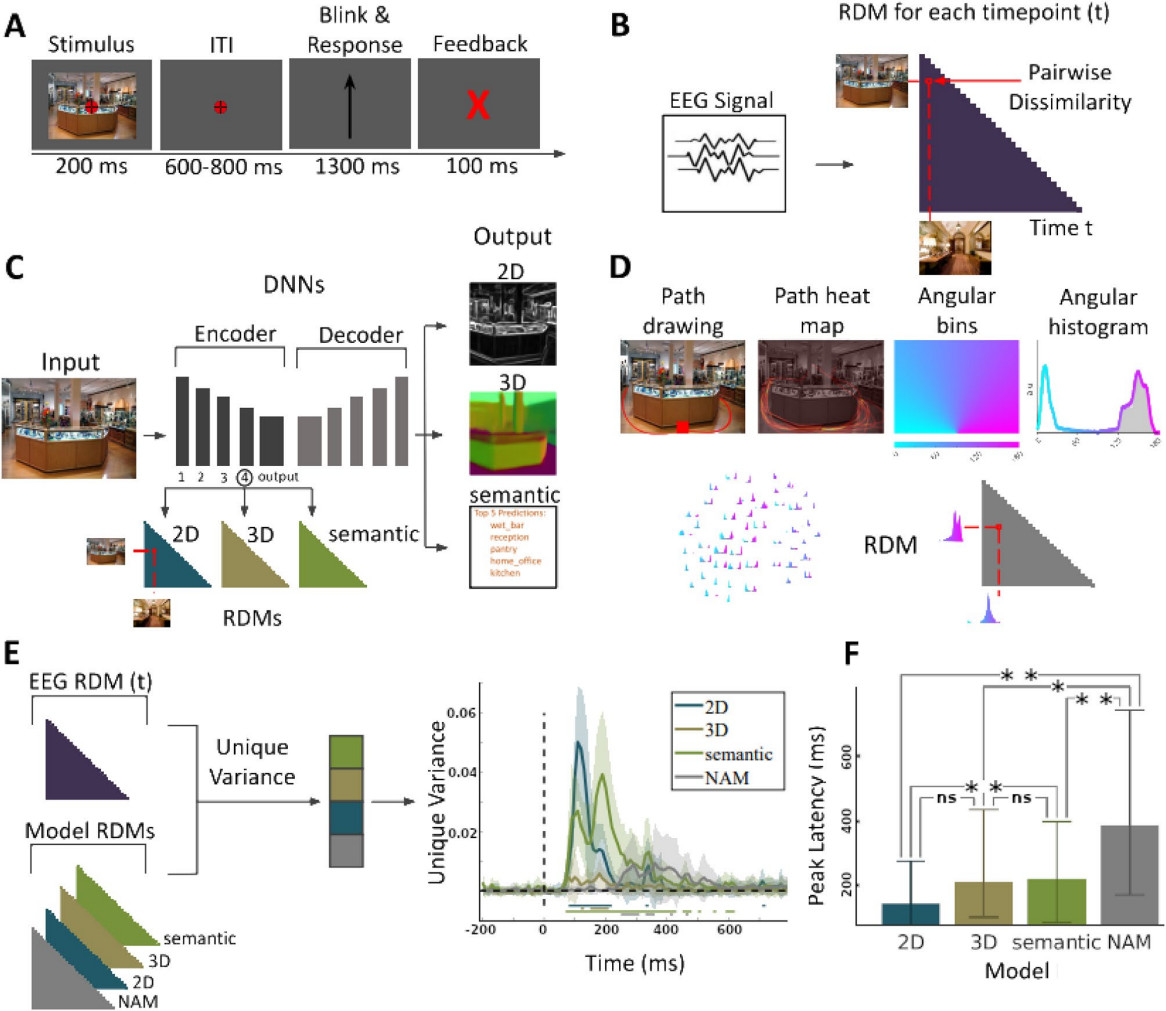
(**A**) EEG paradigm. Participants viewed 50 images of indoor scenes and were asked to mentally plan possible exit paths through the scenes. On interspersed catch trials participants had to respond whether the exit path displayed on the screen corresponded to any of the exit paths from the previous trial. (**B**) EEG RDMs. We computed RDMs for each EEG time point (every 10 ms from − 200 to + 800 ms with respect to image onset). (**C**) DNN RDMs. We calculated RDMs from the activations extracted from the 4th block and output layer of a ResNet50 DNN trained on 2D, 3D and semantic tasks. (**D**) NAM model and RDM^5^. (**E**) Variance partitioning. We calculated the unique EEG variance explained by each of the models, revealing different temporal activation patterns. Lines below the plots indicate significant times using t-test (FDR corrected *p* < 0.05). (**F**) Peak latencies of different models. Error bars indicate the $95\%$ confidence interval. For significance testing we applied bootstrapping followed by FDR correction. We found no significant differences between the correlation peak latency between 2D and 3D models, or 3D and semantic models. However there were significant differences between 2D and semantic models ($p=0.0015$), 2D and NAM models ($p=0.0015$), 3D and NAM models ($p=0.045$), and semantic and NAM models ($P=0.0015$).

We recorded EEG responses from 16 healthy volunteers (7 females, mean age 28.9 ± SD 5.6) to 50 indoor scene images. While viewing the stimuli, participants were asked to assess navigational affordance by imagining the directions of the navigational paths relative to the participant’s viewpoint, i.e., whether the paths were leading to the left, the center, or the right (Fig. 1A).

We then investigated when representations of visual and navigational features emerge in the human brain by comparing the EEG data to deep neural network (DNN) models and behavioral data operationalizing those features using representational similarity analysis.

For this we first transformed the peri-stimulus EEG responses (from − 100 to + 800 ms with respect to stimulus onset) into representational dissimilarity matrices (RDMs) (Fig. 1B) in steps of 10 ms. We then created the 2D, 3D, and semantic RDMs using the activations of DNNs trained on 2D, 3D, and semantic tasks (Fig. 1C).

To construct the navigational affordance model RDM, participants were asked to indicate the exit routes starting from the bottom of a scene image presented to them using a computer mouse. Then, probabilistic heatmaps of navigational affordances were created pooling e data across participants. These heatmaps were transformed into angular histograms that approximate a probabilistic navigational affordance map (NAM) of potential navigational paths radiating from the viewer’s perspective. Pairwise comparison between NAMs resulted in NAM RDMs (Fig. 1D).

Having transformed all the modalities into a common representational space, we performed variance partitioning via regression to find out how much variance of an EEG RDM at a given time point is explained uniquely by the RDMs of any given model (Fig. 1E, left panel). For this, we first performed a regression with all model RDMs as the independent variables and EEG RDM as the dependent variable. This determined the variance explained by all the models together ($R^2_{all}$). Then, we performed a second set of regressions, removing the RDMs of a given model (e.g., NAM) from the independent variables to find the variance explained by models leaving out the model of interest ($R^2_{all-model}$). The unique variance of the EEG RDM explained by the selected model is then calculated as $R^2_{all} - R^2_{all-model}$. For completeness we also plot $R^2_{all}$ and $R^2_{model}$ for each category in Fig. S3.

We found all models explained unique variance in EEG, but to different degrees (Fig. 1E, right panel). The 2D DNN RDM explained the most variance in EEG (max R2 = 0.0502), followed by the semantic DNN (max R2 = 0.0393). The contributions of NAM and 3D DNN RDMs were lower (NAM RDM max R2 = 0.0094, 3D DNN RDM max R2 = 0.0058). This provides evidence that all feature representations can be uniquely tracked in our experiment, and allowed us to inspect the time course further.

We observed a temporal pattern in peak timings (Fig. 1F). The highest uniquely explained variance by the 2D DNN RDM occurred first at 128.12 ± 3.56 ms after stimulus onset, followed by 3D and semantic DNN RDMs peaking at 171.87 ± 30.79 ms and 161.87 ± 10.45 ms, respectively. The unique variance of the NAM RDM reached its peak significantly later than the other model RDMs at 296.25 ± 37.05 ms after stimulus onset. This suggests a hierarchy of scene feature processing leading up to the representation of navigational affordances. A supplementary analysis assessing the average of participant-specific peak latencies rather than the peak latency of the average yielded equivalent results (Supplementary Fig. S2), strengthening our conclusions.

## Discussion

In this study, we investigated the temporal dynamics of scene perception, focusing particularly on the temporal order in which 2D, 3D, semantic features and navigational affordances emerge. We found that the emergence of 2D, semantic and 3D features preceded the emergence of navigational affordance representations.

The early emergence of low-level 2D features followed by high-level semantic features has been previously observed^12^, in particular in studies investigating the correspondence of layers of DNNs trained on scene classification tasks with time-resolved human EEG responses^13^. Equating early layers with low-level visual features and later layers with high-level features yielded a temporal hierarchy, as also observed for object processing^14^. In contrast, 3D features have attracted less attention in M/EEG studies and thus the temporal dynamics with which 3D feature representations emerge are less well understood. Although some studies investigated the temporal dynamics of spatial layout^15,16^, spatial layout represents only coarse-grained 3D features such as the size of the scene in the real world and the position of large surfaces, but does not take into account fine-grained 3D information such as the pose of different objects present in the scene. fMRI studies have investigated fine-grained spatial 3D features by investigating the representation of surface normals^17^ and correspondence to DNNs trained to solve 3D tasks (e.g. depth, occlusion) on the Taskonomy dataset^18,19^ . Here we complement these efforts in the temporal dimension by showing that 3D features are processed in parallel with semantic features.

Navigational affordance representation emerged significantly later than 2D, 3D, and semantic representations. This suggests by temporal order that humans leverage those features to process navigational affordances. In contrast to our results, a recent study^7^ reported both early and late physiological markers of navigational affordances. We note the key differences in both the studies that may have led to different conclusions about navigational affordance processing. First, the images used here are natural and complex, while in^7^ the images were simple and synthetic. Image complexity can influence processing time, potentially due to recurrence^20^. Second, in our study, participants were tasked to identify and find their way around obstacles, making them process occlusions and 3D scene information. In Harel et al.^7^, subjects had to count the number of doors, for which processing 3D scene information might not be needed. The difference in timing might thus reflect a difference in feature processing as required by the task.

A limitation on the ecological validity of our study is that we used static images to assess temporal aspects of scene perception, whereas in real-world situations, humans would process moving images, especially while navigating through indoor environments. Another aspect that could be addressed in the future is inclusion of additional computational models in studying the temporal dynamics of scene perception. Nevertheless, our findings demonstrate a timeline of hierarchical scene feature processing, suggesting that the visual scene features investigated here support navigational planning.

## Methods

### Experiment

#### Participants

We recorded EEG data from 16 healthy volunteers (7 females, mean age 28.9 ± SD 5.6). All participants had normal or corrected-to-normal vision. Participants gave informed consent before the experiment and were provided with monetary compensation. The experiment was conducted in compliance with the Declaration of Helsinki and was approved by the ethics committee of the Freie Universität Berlin.

#### Stimuli

The stimuli were 50 color images of different indoor environments with easily detectable navigational paths originating at the bottom center of each image, previously used in a study by Bonner and Epstein^5^. The dimensions of all the images were 1024 $\times$ 768 pixels and subtended 7^∘^ of visual angle in width and 5.25^∘^ in height. They were presented on a gray screen with a combination of bull’s-eye and crosshair fixation targets^21^ positioned centrally.

#### Experimental paradigm

The paradigm was designed to engage the participants in explicit navigational affordance processing of every image. While viewing the stimuli, participants were asked to imagine the directions of the navigational paths relative to the participant’s viewpoint, i.e., whether the paths were leading to the left, the center, or the right (Fig. 1A).

On each trial Images were presented for 200 ms followed by a randomly varying inter-trial interval between 600 and 800 ms. We chose a 200 ms presentation time in order to avoid eye movements and resulting artifacts in the EEG signal. Image presentation trials were ordered in blocks of one to 5 trials in length.

Blocks were followed by the presentation of a catch trial during which participants had to conduct a task meant to ensure that participants remained attentive and processed the images with respect to spatial and navigational aspects. During catch trials an arrow appeared on the screen for 1.3 s, during which the participants had to indicate whether an arrow on the screen pointed in the same (congruent) or in a different (incongruent) direction than the navigational path in the previous trial. Participants had to respond by pressing the right arrow key for “yes” (congruent, pointing in the same direction) and the left arrow key for “no” (incongruent, not pointing in the same direction). After the response, feedback was presented for 0.1 s, followed by a post-feedback time of 0.2 s. The number of congruent and incongruent trials was balanced across the experiment.

Blocks were organized in runs: there were 69 blocks in total (24 blocks of 5 trials, 15 blocks of 3 and 15 blocks of 4 trials, 10 blocks of 2 trials, and 5 of 1 trial) for each run presented in random order. There were 15 runs (6.2 minutes each) in total in the experiment. This design resulted in each image being repeated 75 times.

### Behavioral data

The behavioral task was designed to be demanding and required participants to engage with the stimuli. Due to technical issues the behavioral data was lost during the EEG acquisition. To determine whether in principle participants perform well on the task, we ran an independent behavioral experiment with 10 additional participants (3 female) using the same paradigm. The average accuracy was $76\%$ (standard deviation = $7.2\%$) which is strongly above the chance level (one tailed t-test, $p = 0.003$). This shows that participants in the additional behavioral sample performed well on the task, suggesting that the task is indeed engaging and could be performed well under the conditions in the EEG experiment.

### EEG

#### EEG recording and pre-processing

For all participants, we recorded continuous neural activity with EEG using the Easycap 64-channel standard electrode system and Brainvision actiCHamp amplifier. We followed the 10-10 system for electrode placement. EEG signals were recorded with a sampling rate of 1000 Hz and bandpass filtered online between 0.03 and 100 Hz.

All electrodes were online referenced to the FCz electrode and grounded to the AFz electrodes. Pre-processing was done offline using FieldTrip^22^. Following previous studies that demonstrated that navigational affordance processing happens in the visual cortex^5^ we selected the 17 most posterior and occipital channels for further analysis (O1, Oz, O2, PO7, PO3, POz, PO4, PO8, P7, P5, P3, P1, Pz, P2, P4, P6, P8). We segmented the recordings into epochs of − 0.2 s to 0.8 s relative to stimulus onset to capture the ERP, baseline-corrected the data to the average pre-stimulus signal for each trial, and then down-sampled the data to 100 Hz. We identified eye blinks and other artifacts with independent component analysis using ICLabel and manual inspection before removal.

#### Pairwise decoding

To determine how well ERP epochs can be used to differentiate between the 50 scene images, we calculated the pairwise decoding accuracy score for each image pair at every time point using CoSMoMVPA^23^. This was done in a time-resolved manner, assessing 100 time points every 0.01s from − 0.2 to 0.8 s relative to image onset. For every possible pair of image conditions, we partitioned the pre-processed ERP epochs across all trial repetitions into training and test data using a leave-one-trial-out cross-validation scheme. We then trained LDA classifiers on all-but-one trials and tested them on the left-out trials. Decoding accuracy scores were averaged across cross-validation folds. To create a grand average time series of EEG decoding accuracy, we calculated the mean decoding accuracy across all pairs.

### Navigational affordance model

To quantify the navigational affordance features in the 50 experimental images, we used the navigational affordance model (NAM) by Bonner and Epstein^5^, which was created using the same set of 50 images. Bonner and Epstein asked participants to draw all possible navigational paths in each image starting from the bottom center of the image. The responses of the participants were aggregated together into heatmaps. Then angular binning was performed to create a navigational affordance histogram by counting the number of pixels in each one-degree bin from 0^∘^ to 180^∘^.

Bonner and Epstein used the behavioral responses to calculate a navigational affordance representational dissimilarity matrix (RDM)^5^. RSA analysis with fMRI recordings revealed affordance representations in the occipital place area. We utilize the same quantification of navigational affordance (via the NAM RDM) to explore when, rather than where, affordance representations emerge.

### Deep neural network models

To assess low-, mid-, and high-level features of indoor scenes, we used activations from 18 pre-trained deep neural network (DNN) models from the Taskonomy Task Bank^10^. The Task Bank consists of DNN that cover various computer vision tasks. We follow previous research demonstrating that the chosen subset of 18 tasks cluster into three categories based on the similarity of the features learned by the DNNs performing the tasks^24^. We refer to those three categories as 2D, 3D, and semantic tasks based on the following rationale; Models from the 2D task category process low-level visual features. Models from the 3D category process such mid-level features as surface normals and depth. Finally models from the semantic category are trained to process high-level semantic features.

Importantly, each of the three groups instantiates a hypothesis about how 2D, 3D and semantic information are represented in the brain that we test by relating model to human brain representations. Our choice of these models and groups as potential models of human brain representations is based on previous research demonstrating that representations in each chosen category (i.e. 2D, 3D and semantic) are related to human brain regions investigated with fMRI with a meaningful and expected pattern^18^. Specifically, while DNNs that process 2D features correlate with brain activations in early visual cortex areas, the unique variance explained for DNNs that process 3D and semantic features was greater in dorsal and ventral areas respectively.

All models were trained on the Taskonomy dataset^10^, which consists of 4.5 million fully annotated images of indoor environments from 600 buildings. We selected the same 18 models of the Taskonomy Task Bank explored in previous research^18^, and completely excluded the remaining 7 models as they did not fit into either of the categories and were thus ill defined for our experimental purpose. In total, there were 7 2D models (trained to perform autoencoding, colorization, denoising, 2D edge detection, inpainting, 2D keypoint detection, and 2D segmentation), 8 3D models (curvature estimation, 3D edge detection, 3D keypoint detection, reshading, euclidean depth prediction, z-buffer depth prediction, surface Normal Estimation, and 2.5D segmentation), and 3 segmentation models (trained to classify objects, places, or perform semantic segmentation). All Taskonomy Test Bank model architectures consist of an encoder and a decoder. The models have identical architecture in the encoder part, and are trained on identical data. They differ only in the decoder architecture (not used here) and the task trained on. Comparing the fit of the models against the brain thus isolates the effect of task on the fitting independent of other factors such as training material and architecture. The encoder architecture is based on ResNet-50^25^ with a compressed convolutional output layer and is identical across the 18 task models. Following^18,24^, to ensure comparability across models, we selected the block4 and the output layer from the identical encoder architecture as the representative task-specific layers for each model.

### EEG-DNN/model comparison

To compare the EEG responses with the DNN and behavioral responses we used representational similarity analysis^26^. In RSA, data from different incommensurate sources are related using a common summary of the representational geometry of each source, enabling unified analysis of data from computational models, behavior, and different neuro-imaging modalities. For this we first computed representational dissimilarity matrices (RDMs) for each model and for the EEG data. RDMs are diagonally symmetric square NxN dimensional matrices (where N is the number of conditions) that summarize the dissimilarity between condition-specific responses in each source space.

Then, we performed a variance partitioning analysis^27^ to estimate the unique variances of the EEG RDMs explained by the model RDM investigated in this work. We detail the RDM construction below.

#### EEG RDMs

We used pairwise decoding accuracy between image conditions to construct EEG RDMs. The rationale is that the more dissimilar the ERP epochs arising from two different images are, the higher the decoding accuracy score for that pair of images will be. RDMs were constructed in a time-resolved manner for each time point (Fig. 1B), yielding 50 $\times$ 50 EEG RDM for each of the 100 time points per participant.

#### Model RDMs

The NAM RDM was constructed by computing the euclidean distance between navigational affordance histograms of all pairs of images (Fig. 1D). We downloaded the precomputed NAM RDMs from https://figshare.com/s/5ff0a04c2872e1e1f416.

For each DNN RDM, we selected block4 and the encoder output layer for creating RDMs. We measured the dissimilarity between any two image representations in the DNN by calculating 1 minus the Pearson correlation distance (1-$\rho$) between the corresponding layer activations. This resulted in two 50 $\times$ 50 RDMs for each DNN (i.e., the block4 and the output layer RDM). RDMs were aggregated and averaged by DNN group for each task type (2D, 3D, semantic) (Fig. 1C), resulting in two RDMs for each task type.

#### Variance partitioning analysis

Since the DNNs investigated are trained on the same dataset, their RDMs are expected to be correlated. To nevertheless identify the aspects unique to a particular task type, we use variance partitioning with the goal to identify variance uniquely attributable to any one model. To compute the unique variance of a given model, we calculated the difference in variance explained when all the model RDMs are used as independent variables and variance explained when all but current model RDMs are used as independent variables.

We conducted this analysis at every time point separately, i.e. every 10 ms from − 200 to + 800 ms relative to image onset. In the regression we used the lower triangular part of the RDM as it describes the representational geometry fully and avoids potential artifacts created by including the diagonal. This resulted in four time series per participant, one for each model (3D, 3D, semantic, navigational affordance) indicating when feature representations corresponding to the model type emerge during visual processing (See Fig. S1). The averaged unique variance plots are presented in Fig. 1E.

#### Statistical analysis

We used bootstrapping with 1000 iterations to assess the statistical significance of the unique variance explained by different models and participant-specific peak latencies. We corrected the *p*-values for multiple comparisons by applying FDR correction with a threshold of 0.05. Supplementary analysis assessing the average of participant-specific peak latencies using a Welch T-test yielded equivalent results (See Fig. S2).

### Supplementary Information

Supplementary Information.

## Data Availability

The code and data necessary for reproducing the results presented in this paper can be found at https://osf.io/wz4ha/.

## References

[1] Fei-Fei L, Iyer A, Koch C, Perona P (2007). What do we perceive in a glance of a real-world scene?. J. Vis..

[2] Greene MR, Oliva A (2009). The briefest of glances: The time course of natural scene understanding. Psychol. Sci..

[3] Potter MC (1975). Meaning in visual search. Science.

[4] Thorpe S, Fize D, Marlot C (1996). Speed of processing in the human visual system. Nature.

[5] Bonner MF, Epstein RA (2017). Coding of navigational affordances in the human visual system. Proc. Natl. Acad. Sci..

[6] Djebbara, Z., Fich, L. B., Petrini, L. & Gramann, K. Sensorimotor brain dynamics reflect architectural affordances. In *Proceedings of the National Academy of Sciences*, Vol. 116 14769–14778. 10.1073/pnas.1900648116https://www.pnas.org/doi/pdf/10.1073/pnas.1900648116 (2019). 10.1073/pnas.1900648116 PMC6642393 31189596

[7] Harel A, Nador JD, Bonner MF, Epstein RA (2022). Early electrophysiological markers of navigational affordances in scenes. J. Cogn. Neurosci..

[8] Kalénine S, Wamain Y, Decroix J, Coello Y (2016). Conflict between object structural and functional affordances in peripersonal space. Cognition.

[9] Mustile M, Giocondo F, Caligiore D, Borghi AM, Kourtis D (2021). Motor inhibition to dangerous objects: Electrophysiological evidence for task-dependent aversive affordances. J. Cogn. Neurosci..

[10] Zamir, A. R. *et al.* Taskonomy: Disentangling task transfer learning. In *Proceedings of the IEEE Conference on Computer Vision and Pattern Recognition* 3712–3722 (2018).

[11] Bonner MF, Epstein RA (2018). Computational mechanisms underlying cortical responses to the affordance properties of visual scenes. PLoS Comput. Biol..

[12] Harel A, Groen IIA, Kravitz DJ, Deouell LY, Baker CI (2016). The temporal dynamics of scene processing: A multi-faceted EEG investigation. ENeuro.

[13] Greene MR, Hansen BC (2018). Shared spatiotemporal category representations in biological and artificial deep neural networks. PLoS Comput. Biol..

[14] Cichy RM, Khosla A, Pantazis D, Torralba A, Oliva A (2016). Comparison of deep neural networks to spatio-temporal cortical dynamics of human visual object recognition reveals hierarchical correspondence. Sci. Rep..

[15] Cichy RM, Khosla A, Pantazis D, Oliva A (2017). Dynamics of scene representations in the human brain revealed by magnetoencephalography and deep neural networks. Neuroimage.

[16] Henriksson L, Mur M, Kriegeskorte N (2019). Rapid invariant encoding of scene layout in human OPA. Neuron.

[17] Lescroart MD, Gallant JL (2019). Human scene-selective areas represent 3D configurations of surfaces. Neuron.

[18] Dwivedi K, Bonner MF, Cichy RM, Roig G (2021). Unveiling functions of the visual cortex using task-specific deep neural networks. PLoS Comput. Biol..

[19] Wang AY, Wehbe L, Tarr MJ (2019). Neural taskonomy: Inferring the similarity of task-derived representations from brain activity. Adv. Neural Inf. Process. Syst..

[20] Kar K, Kubilius J, Schmidt K, Issa EB, DiCarlo JJ (2019). Evidence that recurrent circuits are critical to the ventral stream’s execution of core object recognition behavior. Nat. Neurosci..

[21] Thaler L, Schütz AC, Goodale MA, Gegenfurtner KR (2013). What is the best fixation target? The effect of target shape on stability of fixational eye movements. Vis. Res..

[22] Oostenveld R, Fries P, Maris E, Schoffelen J-M (2010). FieldTrip: Open source software for advanced analysis of MEG, EEG, and invasive electrophysiological data. Comput. Intell. Neurosci..

[23] Oosterhof NN, Connolly AC, Haxby JV (2016). CoSMoMVPA: Multi-modal multivariate pattern analysis of neuroimaging data in Matlab/GNU Octave. Front. Neuroinform..

[24] Dwivedi, K. & Roig, G. Representation similarity analysis for efficient task taxonomy and transfer learning. In *Proceedings of the IEEE/CVF Conference on Computer Vision and Pattern Recognition (CVPR)* (2019).

[25] He, K., Zhang, X., Ren, S. & Sun, J. Deep residual learning for image recognition. In *2016 IEEE Conference on Computer Vision and Pattern Recognition (CVPR)* 770–778. 10.1109/CVPR.2016.90 (2016).

[26] Kriegeskorte N, Mur M, Bandettini PA (2008). Representational similarity analysis-connecting the branches of systems neuroscience. Front. Syst. Neurosci..

[27] Legendre P (2008). Studying beta diversity: Ecological variation partitioning by multiple regression and canonical analysis. J. Plant Ecol..

[28] Bennett, L., Melchers, B. & Proppe, B. Curta: A general-purpose high-performance computer at ZEDAT, Freie Universität Berlin. 10.17169/refubium-26754 (2020).

