# Supplementary material for: Visual features are processed before navigational affordances in the human brain
Source: Sci Rep. 2024 Mar 6;14:5573. doi: 10.1038/s41598-024-55652-y (PMC10917749; doi:10.1038/s41598-024-55652-y)
Supplement: Supplementary file 1 — Supplementary Information. [file 41598_2024_55652_MOESM1_ESM.pdf]

Supplementary Material: Visual features are processed before navigational affordances in the human brain

1 Individual Subject Correlation

Unique variance per participant and model category. The plot in Figure 1F is the average for all 16 subjects. Values plotted in Figure S2 are the averages of the latency of the absolute peak for each of individual participant.

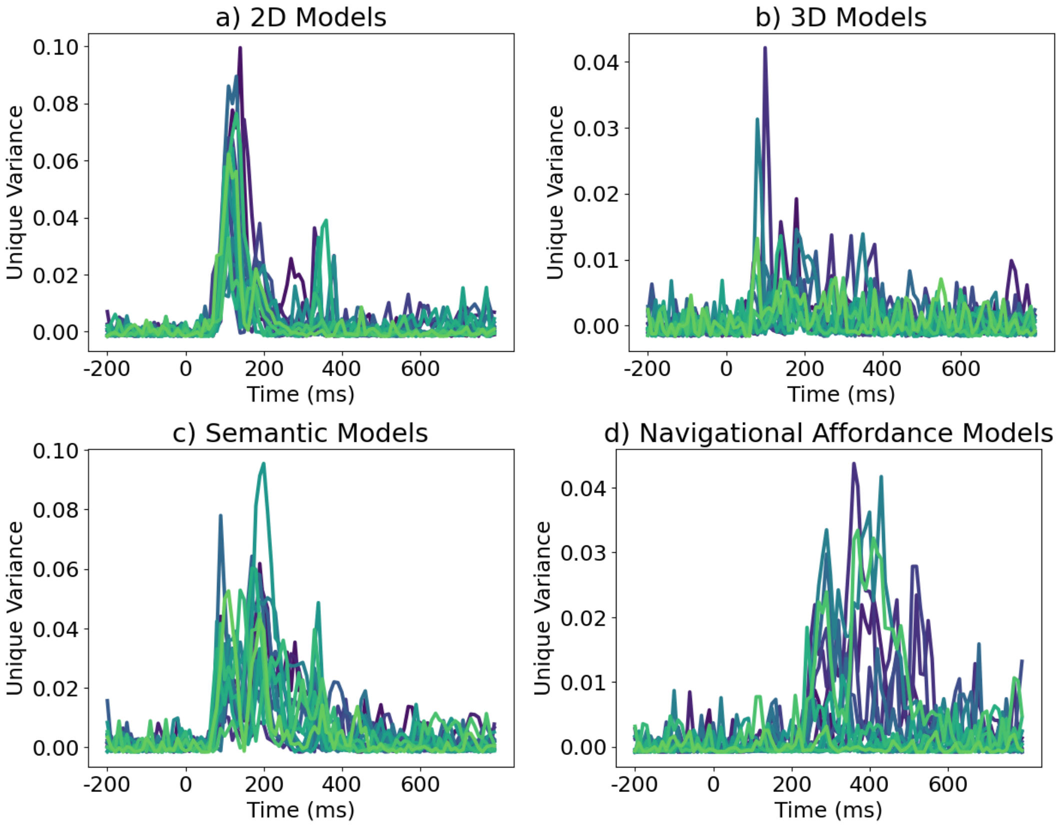

Figure S1 Unique variance plotted for the a) 2D model, b) 3D model, c) the semantic model and d) Navigational Affordance model (NAM). Individual lines represent individual participants.

2 Peak Latency Analysis

Welch T-tests analysis of peak latencies. The peaks were calculated for each subject and then averaged. Significant correlations were tested using unequal variances Welch T-test. As can be observed, we receive the same pattern of results as we did when using bootstrapping.

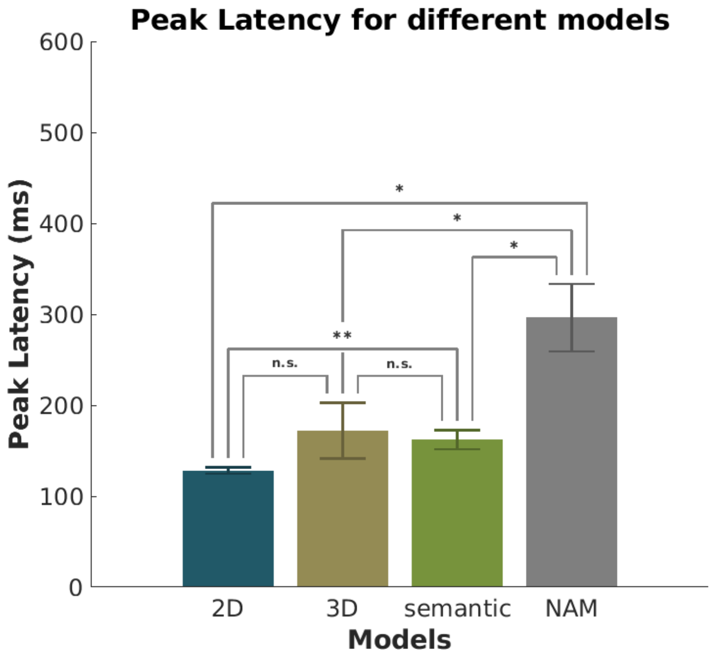

Figure S2 Peak latencies of different models. Error bars indicate standard deviation for 16 subjects. For significance testing we applied two-tailed Welch T-tests followed by FDR correction. We found no significant differences between the correlation peak latency between 2D and 3D models, or 3D and semantic models (p=0.2 and p=0.7). However there were significant differences between 2D and semantic models (p=0.004), 2D and NAM models (p=0.028), 3D and NAM models (p=0.021), and semantic and NAM models (p=0.021).

3 Model Correlations

To put the quality of the results in Fig. 1F into context, we calculated the R^2 (total variance explained) using models from each category, as well as R^2_{all}. Additionally we also calculated the upper and lower bounds of the noise ceiling (indicated by the shaded grey region). Given N subjects, the lower noise ceiling is estimated at each time point by calculating the R^2 of each individual subject RDM with the mean RDM of the other $(N-1)$ subjects. The upper noise ceiling is estimated in a similar fashion while using the mean RDM of all the subjects (N) instead. The N R^2 values are averaged to get the lower and upper noise ceiling bounds.

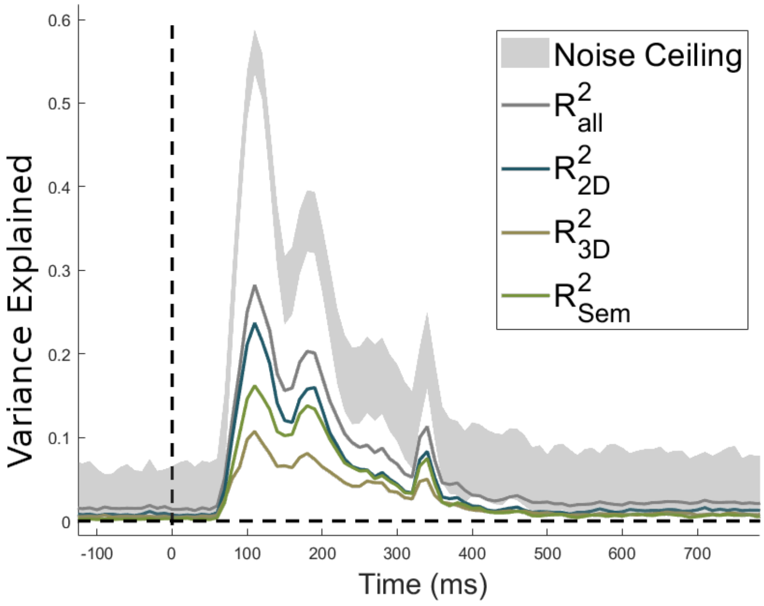

Figure S3 The overall correlations using all model RDMs, only 2D model RDMs, and 3D model RDMs. The upper and lower bounds of the noise ceilings are represented by the shaded gray area.

4 Taskonomy Model Selection

The Taskonomy model bank[1] consists of 25 models with an identical ResNet-50 encoder architecture trained to perform various visual tasks. Previous research demonstrated that most of the models can be divided into those solving 2D, 3D, and semantic tasks. Within each of these three categories the models learn similar representations, enabling more efficient transfer learning[2]. Moreover, variance partitioning analysis also demonstrated that the representations learned by the 2D, 3D, and Semantic models correlated with early, dorsal, and ventral visual cortex regions[3].

We used the same models and categorized them in the same way as previous research[2,3]. In detail, models in the 2D category were trained to perform the following tasks: autoencoding, colorization, denoising, 2D edge detection, inpainting, 2D keypoint detection, and 2d segmentation. Models in the 3D category were trained to perform curvature estimation, 3d edge detection, 3d keypoint detection, reshading, depth prediction (rgb2depth and rgb2mist), surface Normal Estimation, and 2.5D segmentation. Finally, Semantic models were trained to classify objects, classify places, or perform semantic segmentation.

References

1. Zamir, A. R. *et al.* Taskonomy: Disentangling task transfer learning. *Proc. IEEE conference on computer vision pattern recognition* 3712–3722. (2018).

2. Dwivedi, K. & Roig, G. Representation similarity analysis for efficient task taxonomy and transfer learning. In *Proceedings of the IEEE/CVF Conference on Computer Vision and Pattern Recognition (CVPR)* (2019).

3. Dwivedi, K., Bonner, M. F., Cichy, R. M. & Roig, G. Unveiling functions of the visual cortex using task-specific deep neural networks. *PLOS Comput. Biol.* **17(8), e1009267.**, DOI: https://doi.org/10.1371/journal.pcbi.1009267 (2021).
